# Development and Validation of a Predictive Nomogram for Possible REM Sleep Behavior Disorders

**DOI:** 10.3389/fneur.2022.903721

**Published:** 2022-06-29

**Authors:** Hong Lai, Xu-Ying Li, Junya Hu, Wei Li, Fanxi Xu, Junge Zhu, Raoli He, Huidan Weng, Lina Chen, Jiao Yu, Xian Li, Yang Song, Xianling Wang, Zhanjun Wang, Wei Li, Rong Kang, Yuling Li, Junjie Xu, Yuanfei Deng, Qinyong Ye, Chaodong Wang

**Affiliations:** ^1^Department of Neurology, National Clinical Research Center for Geriatric Diseases, Xuanwu Hospital of Capital Medical University, Beijing, China; ^2^Institute of Genetics and Developmental Biology, Chinese Academy of Sciences, Beijing, China; ^3^Department of Neurobiology, National Clinical Research Center for Geriatric Diseases, Xuanwu Hospital of Capital Medical University, Beijing, China; ^4^Department of Neurology, Fujian Key Laboratory of Molecular Neurology, Fujian Medical University Union Hospital, Institute of Neuroscience, Fujian Medical University, Fuzhou, China; ^5^The Xinjiekou Community Health Service Center, Beijing, China; ^6^The Qinglonghu Community Health Service Center, Beijing, China; ^7^Department of Geriatric Disease, Peking University Shenzhen Hospital, Shenzhen, China

**Keywords:** REM sleep behavior disorder (RBD), LASSO, nomogram, decision curve analysis (DCA), predictive model

## Abstract

**Objectives:**

To develop and validate a predictive nomogram for idiopathic rapid eye movement (REM) sleep behavior disorder (RBD) in a community population in Beijing, China.

**Methods:**

Based on the validated RBD questionnaire-Hong Kong (RBDQ-HK), we identified 78 individuals with possible RBD (pRBD) in 1,030 community residents from two communities in Beijing. The least absolute shrinkage and selection operator (LASSO) regression was applied to identify candidate features and develop the nomogram. Internal validation was performed using bootstrap resampling. The discrimination of the nomogram was evaluated using the area under the curve (AUC) of the receiver operating characteristic (ROC) curve, and the predictive accuracy was assessed *via* a calibration curve. Decision curve analysis (DCA) was performed to evaluate the clinical value of the model.

**Results:**

From 31 potential predictors, 7 variables were identified as the independent predictive factors and assembled into the nomogram: family history of Parkinson's disease (PD) or dementia [odds ratio (OR), 4.59; 95% confidence interval (CI), 1.35–14.45; *p* = 0.011], smoking (OR, 3.24; 95% CI, 1.84–5.81; *p* < 0.001), physical activity (≥4 times/week) (OR, 0.23; 95% CI, 0.12–0.42; *p* < 0.001), exposure to pesticides (OR, 3.73; 95%CI, 2.08–6.65; *p* < 0.001), constipation (OR, 6.25; 95% CI, 3.58–11.07; *p* < 0.001), depression (OR, 3.66; 95% CI, 1.96–6.75; *p* < 0.001), and daytime somnolence (OR, 3.28; 95% CI, 1.65–6.38; *p* = 0.001). The nomogram displayed good discrimination, with original AUC of 0.885 (95% CI, 0.845–0.925), while the bias-corrected concordance index (C-index) with 1,000 bootstraps was 0.876. The calibration curve and DCA indicated the high accuracy and clinical usefulness of the nomogram.

**Conclusions:**

This study proposed an effective nomogram with potential application in the individualized prediction for pRBD.

## Introduction

Rapid eye movement (REM) sleep behavior disorder (RBD) is a parasomnia characterized by loss of normal muscle atonia during the REM sleep, presenting with complex behaviors associated with a nightmare (1). The gold standard diagnostic criterion of RBD is video-polysomnography (vPSG), with a video demonstration of complex motor behaviors accompanied by an excess of muscle tone and/or phasic muscle twitching during REM sleep (2). The clinical features of RBD could be variable with a spectrum of dream-enacting behavioral expression from simple acts, such as talking in sleep and shouting to complex body movements that include punching, kicking, and falling out of bed (3), which can lead to significant injuries. These severe symptoms usually prompt patients to seek medical aid.

Idiopathic RBD (iRBD) is by far the strongest risk factor for prodromal synucleinopathies (4), a group of neurodegenerative disorders, such as Parkinson's disease (PD), multiple system atrophy (MSA), and dementia with Lewy bodies (DLB) (5–8). They all have a long prodromal stage in which symptoms of neurodegeneration are perceptible, but the full clinical disease has not developed yet (9). More than 80% of individuals with iRBD will develop one of these neurodegenerative diseases within 20 years of onset of iRBD (6, 10). Because of the strong relationship between iRBD and neurodegenerative diseases, identification of risk factors for iRBD and early detection of individuals who are likely to develop iRBD is of great importance and may aid in delivering the proper intervention and minimizing its injurious potential. Furthermore, patients with RBD can be ideal candidates for screening new neuroprotective methods and a better understanding of the progression and pathophysiology of synucleinopathies from their presymptomatic phases. Some large population-based studies reported the potential risk factors for iRBD (11–15), such as socioeconomic status, male sex, lower education, head injury, pesticide exposure, cigarette smoking, alcohol use, carbon monoxide (CO) poisoning, a family history of PD or dementia, psychological distress, and so on. Considering that vPSG is time and resource consuming and can only be feasible in medical centers, most of the studies used questionnaires as screening tools for iRBD diagnosis.

Although many predictive factors for iRBD have been proposed, none of them has been integrated into a prediction model. The nomogram has been accepted as a reliable tool to create a simple intuitive graph of a statistical predictive model that quantifies the risk of a clinical event (16, 17). Nomograms enable specific individual risk scores by numerical estimation of the possibility of an event that is tailored to the situation of an individual. The use of nomograms is facilitated by user-friendly graphical interfaces for generating the estimates during clinical encounters to inform clinical decision-making (17). The aim of this study was to develop and validate a nomogram for predicting the risk of possible RBD (pRBD), based on two elderly community cohorts in Beijing, China.

## Methods

### Study Design and Data Source

The study was approved by the Medical Ethics Committee of Xuanwu Hospital of Capital Medical University. Informed consent was obtained from all subjects in the study. The present study used a random sampling method to select a community cohort of Beijing residents aged ≥50 years from April 2019 to September 2021. Individuals were selected from 7 community units of one urban district (Xicheng district) and 13 community units of a suburb district (Fangshan district). In total, 1,300 persons were invited, of whom 232 were not eligible, mainly because inclusion criteria were not met (*n* = 133), the persons moved before recruitment (*n* = 39), mobile phone was not connected (*n* = 55), or deceased (*n* = 5), resulting in a final source population of 1,068 persons. Among them, there were 38 individuals who missed data for some critical assessments and, thus, were excluded from the study (Supplementary Figure S1). The response rate of the study was 96.44%. The individuals were excluded from the study using the following criteria: (i) subjects with PD-related motor symptoms (bradykinesia, tremor, postural instability, and rigidity); (ii) diagnosed with dementia or PD and other neurodegenerative diseases; (iii) subjects with malignant tumors or other serious systemic diseases. All the subjects were assessed for demographic information, history of chronic diseases, medication use, lifestyle behaviors, and environmental exposures. Subjects were also assessed for motor and non-motor symptoms of PD, such as RBD status, using scales (additional information was given in Supplementary Material). All the assessments were performed *via* face-to-face interviews by clinical investigators with unified training.

### Candidate Predictors

Based on the previously published literature (11–15), the following 31 potential predictive variables were selected for prediction: demographic variables (age, sex, and education level), body mass index (BMI), family history of PD or dementia, medical history (hypertension, diabetes, coronary heart disease, hyperlipidemia, stroke, head injury with unconsciousness, CO poisoning with unconsciousness, and general anesthesia), medications (such as aspirin, statins, calcium antagonist, and antidiabetics), non-motor symptoms of PD [olfactory dysfunction, constipation, cognitive impairment, depression, daytime somnolence, and scale for outcomes in PD-autonomic (SCOPA-AUT) scores], lifestyle (smoking, drinking of alcohol, tea, coffee, and physical activity), and environmental exposures (pesticides, organic solvents, and heavy metals).

### Outcome Measure

The main outcome was the diagnosis of pRBD using the validated RBD Questionnaire–Hong Kong (RBDQ-HK) (Supplementary Table S1). The RBDQ-HK is a self-administered questionnaire comprising 13 questions related to various clinical features of RBD, which are rated on scales of lifetime occurrence and recent 1-year frequency (18). The questionnaire consists of factor 1 (Q1–Q5, and Q13, dream-related factor) and factor 2 (Q6–Q12, behavioral manifestations factor). The best cutoff score for the overall RBDQ-HK questionnaire was found to be at 18/19, which had good sensitivity (82.2%) and specificity (86.9%) in a large PSG-based study (18). The RBDQ-HK overall scale had a sensitivity of 85% and specificity of 81% in another validation study in East China, which included patients with PD or obstructive sleep apnea (19). Therefore, a score ≥19 for the overall scale of RBDQ-HK was considered to be pRBD status in the study, without further verification by PSG.

### Statistical Analysis

All statistical analyses were performed with the R software (version 4.1.1; R Foundation for Statistical Computing Vienna, Austria; http://www.R-project.org/). Categorical variables were expressed as frequencies and percentages, while continuous variables were described as medians and interquartile ranges (IQRs). Mann-Whitney U tests (for continuous variables with skewness distributions) and the chi-square test (for categorical variables) were utilized to compare the differences in characteristics between groups. The R package “glmnet” was used to perform the least absolute shrinkage and selection operator (LASSO) regression and the R package “rms” to establish the nomogram and calibration curve. The R package “pROC,” “plotROC,” and “rmda” were applied to generate the receiver operating characteristic (ROC) and decision curve analysis (DCA). All tests were two-tailed and *p* < 0.05 was defined as statistically significant (original data and analysis code were given in Supplementary Material).

### Variable Selection and Establishment of a Predictive Model

The least absolute shrinkage and selection operator (LASSO) regression with 10-fold cross-validation was used for the most useful predictive factors from the initial dataset to address multiple cross-related covariates and reduce the risk of overfitting the data (20). It adds an L1 norm as a penalty in the calculation of the minimum residual sum of squares (RSS). As the lambda gets large, some coefficients can be accurately shrunk to zero (20, 21). We then selected the lambda of 1 standard for which the cross-validation error is the smallest. Multivariable logistic regression analysis was performed to develop a predictive model of pRBD risk based on the predictors selected by LASSO. A nomogram was created based on the results of multivariable analyses. Predictors whose *p*-values were <0.05 were included.

### Model Performance

Model performance is divided into two main categories: discriminative ability and model calibration. The discriminative ability assesses whether the model is able to differentiate between patients with a favorable and an unfavorable outcome. It is expressed by the area under the curve (AUC) of a ROC curve (22). Model calibration assesses to what extent predicted values agree with the observed outcomes. It was assessed using a calibration plot, in which predicted probabilities are plotted against observed outcome frequencies (23). For a well-calibrated model, the predictions should fall on a 45-degree diagonal line. Overall concordance between predicted and observed outcomes was tested using the Hosmer-Lemeshow test (23). A *p* > 0.05 was considered as well calibrated.

### Model Validation

Internal validation evaluating the stability of a prediction model to random changes in sample composition was performed by the bootstrap resampling, in which regression models were fitted in 1,000 bootstrap replicates, drawn with replacement from the development sample (24). The model was refitted in each bootstrap replicate and tested on the original sample to estimate optimism in model performance (17). Specifically, a bias-corrected concordance index (C-index) was estimated using a 1,000-sample bootstrap to calculate the discrimination of the model.

### Clinical Utility of Nomogram

The clinical usefulness of the nomogram was determined by DCA by quantifying the net benefit to the subjects under different threshold probabilities (25).

## Results

### Clinical and Demographic Characteristics

Table 1 summarizes the demographic and clinical data of the subjects. The median age of the subjects was 68 years (IQR, 64–72 years). Men accounted for 39.22% and women accounted for 60.78%, and most had received junior to middle school education. After screening using the RBDQ-HK scale, a total of 78 subjects were positive for pRBD. Among all the subjects, 2.04% had a family history of PD or dementia, 57.57% had hypertension, 27.57% had diabetes, 12.62% had coronary heart disease, 6.89% had a stroke, and 38.16% had hyperlipidemia.

**Table 1:** Characteristics of total subjects.

**Variables**	**Total** **(*n* = 1,030)**	**No pRBD** **(*n* = 952)**	**pRBD** **(*n* = 78)**	***p-*** **value**
**Demographics**				
Age, year	68 (64, 72)	68 (64, 72)	69 (65.25, 72)	0.158
<65	344 (33.4)	324 (34.03)	20 (25.64)	0.317
65–75	548 (53.2)	502 (52.73)	46 (59.97)	
>75	138 (13.4)	126 (13.24)	12 (15.38)	
Gender				0.827
Male	404 (39.22)	372 (39.08)	32 (41.03)	
Female	626 (60.78)	580 (60.92)	46 (58.97)	
**Education level**				
Years of education	9 (8, 12)	9 (8, 12)	9 (6.25, 12)	0.593
Illiterate	25 (2.43)	21 (2.21)	4 (5.13)	0.326
Primary school	200 (19.42)	184 (19.33)	16 (20.51)	
Middle school	649 (63.01)	604 (63.45)	45 (57.69)	
High school and above	156 (15.15)	143 (15.02)	13 (16.67)	
BMI, kg/m^2^	24.65 (22.66, 26.95)	24.66 (22.66, 26.95)	24.5 (22.71, 26.57)	0.881
Obesity	76 (7.38)	73 (7.67)	3 (3.85)	0.310
Family history of PD or dementia	21 (2.04)	15 (1.58)	6 (7.69)	0.003^*^
**Lifestyle behaviors**				
Smoking	338 (32.82)	295 (30.99)	43 (55.13)	<0.001^*^
Alcohol	282 (27.38)	247 (25.95)	35 (44.87)	<0.001^*^
Tea	536 (52.04)	494 (51.89)	42 (53.85)	0.830
Coffee	83 (8.06)	80 (8.40)	3 (3.85)	0.228
Physical activity				<0.001^*^
<1 times/week	235 (22.82)	199 (20.9)	36 (46.15)	
<4 times/week	90 (8.74)	78 (8.19)	12 (15.38)	
≥2 times/week	705 (68.45)	675 (70.9)	30 (38.46)	
**Environmental exposure**				
Pesticides	159 (15.44)	121 (12.71)	38 (48.72)	<0.001^*^
Organic solvent	35 (3.40)	31 (3.26)	4 (5.13)	0.331
Heavy metal	30 (2.91)	27 (2.84)	3 (3.85)	0.491
CO poisoning with unconsciousness	103 (10.0)	92 (9.66)	11 (14.10)	0.289
Head injury with unconsciousness	31 (3.01)	27 (2.84)	4 (5.13)	0.286
**Medical history**				
Hypertension	593 (57.57)	543 (57.04)	50 (64.10)	0.274
Diabetes	284 (27.57)	266 (27.94)	18 (23.08)	0.428
Coronary heart disease	130 (12.62)	115 (12.08)	15 (19.23)	0.099
Hyperlipidemia	393 (38.16)	364 (38.24)	29 (37.18)	0.950
Stroke	71 (6.89)	65 (6.83)	6 (7.69)	0.954
General anesthesia	244 (23.69)	228 (23.95)	16 (20.51)	0.584
**Medication use**				
Aspirin	290 (28.16)	266 (27.94)	24 (30.877)	0.687
Statins	427 (41.46)	394 (41.39)	33 (42.31)	0.969
Calcium antagonist	356 (34.56)	326 (34.24)	30 (38.46)	0.529
Antidiabetics	271 (26.31)	253 (26.58)	18 (23.08)	0.589
**Non-motor symptoms**				
Olfactory dysfunction	143 (13.88)	129 (13.55)	14 (17.95)	0.363
Constipation	197 (19.13)	150 (15.76)	47 (60.26)	<0.001^*^
Cognitive impairment	63 (6.12)	54 (5.67)	9 (11.54)	0.048^*^
Depression	128 (12.43)	100 (10.50)	28 (35.90)	<0.001^*^
Daytime somnolence	109 (10.58)	85 (8.93)	24 (30.77)	<0.001^*^
SCOPA-AUT scores	3 (1, 6)	3 (1, 6)	4.5 (1.25, 7)	0.039^*^

*pRBD, possible REM sleep behavior disorder; BMI, body mass index; CO, carbon monoxide; SCOPA-AUT, scale for outcomes in PD-autonomic*.

*Values are n (%) or medians and interquartile ranges (IQRs)*.

* *p < 0.05 was defined as statistically significant*.

Subjects with pRBD were more probable to report a family history of PD or dementia than subjects without pRBD and were more likely to be smokers or alcohol drinkers when compared with subjects without pRBD. Subjects with pRBD had a lower frequency of physical activity than those without. We found a significantly higher occurrence of exposure to pesticides in subjects with pRBD than in those without. We also found an increased prevalence of constipation, depression, and daytime somnolence among subjects with pRBD. On the SCOPA-AUT scores, subjects with pRBD had a higher score than those without. No statistically significant differences were found in other factors between the subjects with and without pRBD.

### Variable Selection and Model Construction

**Figure 1:**
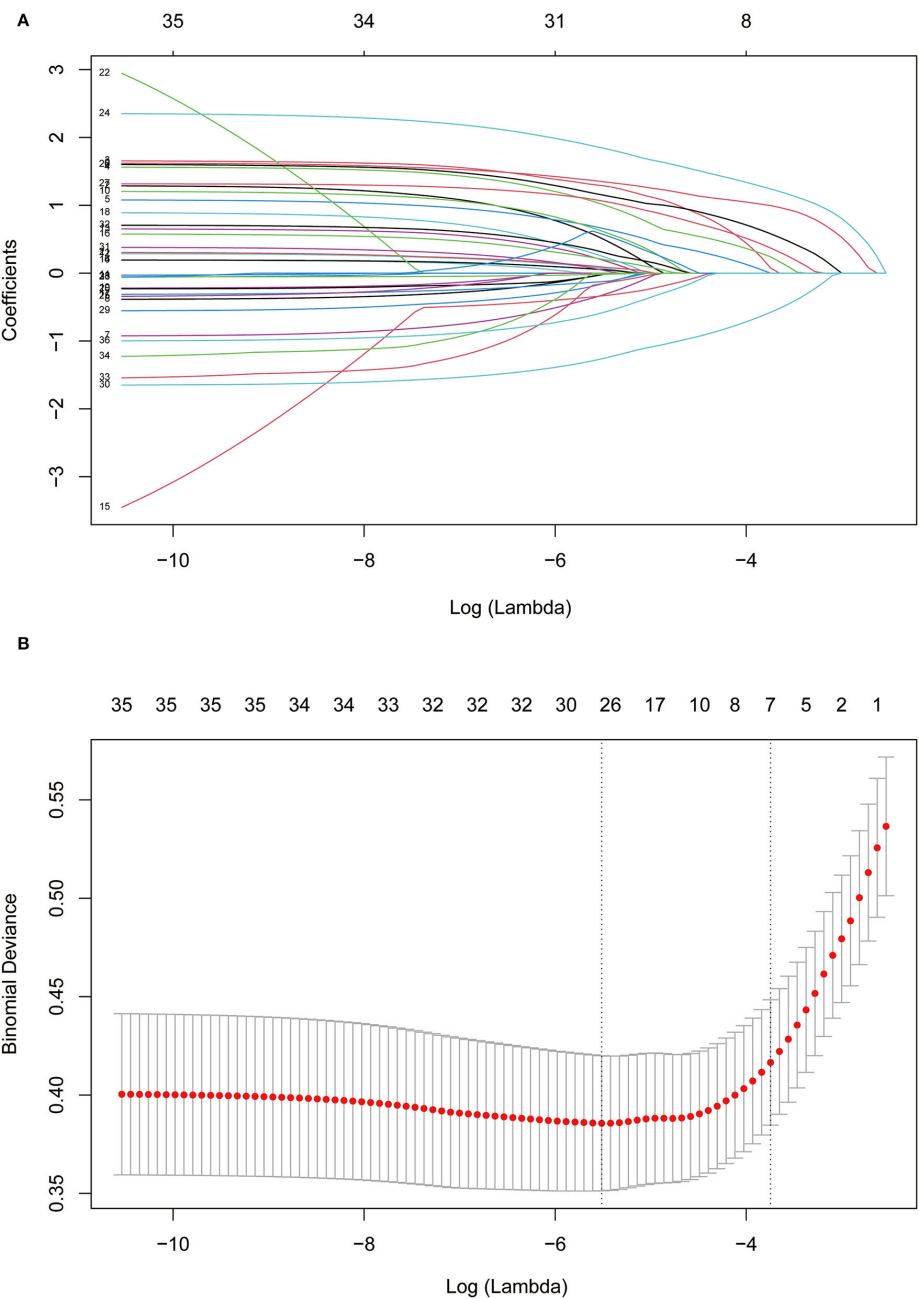
Feature selection using Lasso regression. **(A)** Lasso coefficient profiles of the candidate features. **(B)** The selection of optimal parameters (lambda) by 10-fold cross-validation. The left and right dotted vertical lines, respectively, represented the optimal lambda values when using the minimum error criterion and one standard error (1-SE) of the minimum criterion.

A LASSO regression model with 10-fold cross-validation was employed to select predictive variables among the preliminarily screened factors (Table 1). A total of 31 variables assessed at baseline were included in the LASSO regression. After LASSO regression selection (Figure 1), 7 variables remained to be non-zero coefficients that minimized the overall Lambda and were confirmed as the potentially optimal variables for predicting pRBD, such as a family history of PD or dementia, smoking, physical activity, exposure to pesticides, constipation, depression, and daytime somnolence.

Inclusion of these 7 variables in a multivariate logistic regression model resulted in all the 7 variables that were independently statistically significant predictors for pRBD. These variables included family history of PD or dementia [odds ratio (OR), 4.59; 95% confidence interval (CI), 1.35–14.45; *p* = 0.011], smoking (OR, 3.24; 95% CI, 1.84–5.81; *p* < 0.001), physical activity (≥4 times/week) (OR, 0.23; 95% CI, 0.12–0.42; *p* < 0.001), exposure to pesticides (OR, 3.73; 95% CI, 2.08–6.65; *p* < 0.001), constipation (OR, 6.25; 95% CI, 3.58–11.07; *p* < 0.001), depression (OR, 3.66; 95% CI, 1.96–6.75; *p* < 0.001), and daytime somnolence (OR, 3.28; 95% CI, 1.65–6.38; *p* = 0.001; Table 2). Using these variables, the nomogram was constructed.

**Table 2:** Parameters selected for multivariate logistic regression models for predicting possible REM sleep behavior disorder (pRBD).

	**Odds ratio (95% CI)**	***p*** **-value**
Family history of PD or dementia	4.59 (1.35, 14.45)	0.011^*^
Smoking	3.24 (1.84, 5.81)	<0.001^*^
Pesticides	3.73 (2.08, 6.65)	<0.001^*^
Physical activity (≥4 times/week)	0.23 (0.12, 0.42)	<0.001^*^
Constipation	6.25 (3.58, 11.07)	<0.001^*^
Depression	3.66 (1.96, 6.75)	<0.001^*^
Daytime somnolence	3.28 (1.65, 6.38)	0.001^*^

*CI, confidence interval*.

* *p < 0.05 was defined as statistically significant*.

### Performance of the Nomograms and Bootstrap Internal Validation

**Figure 2:**
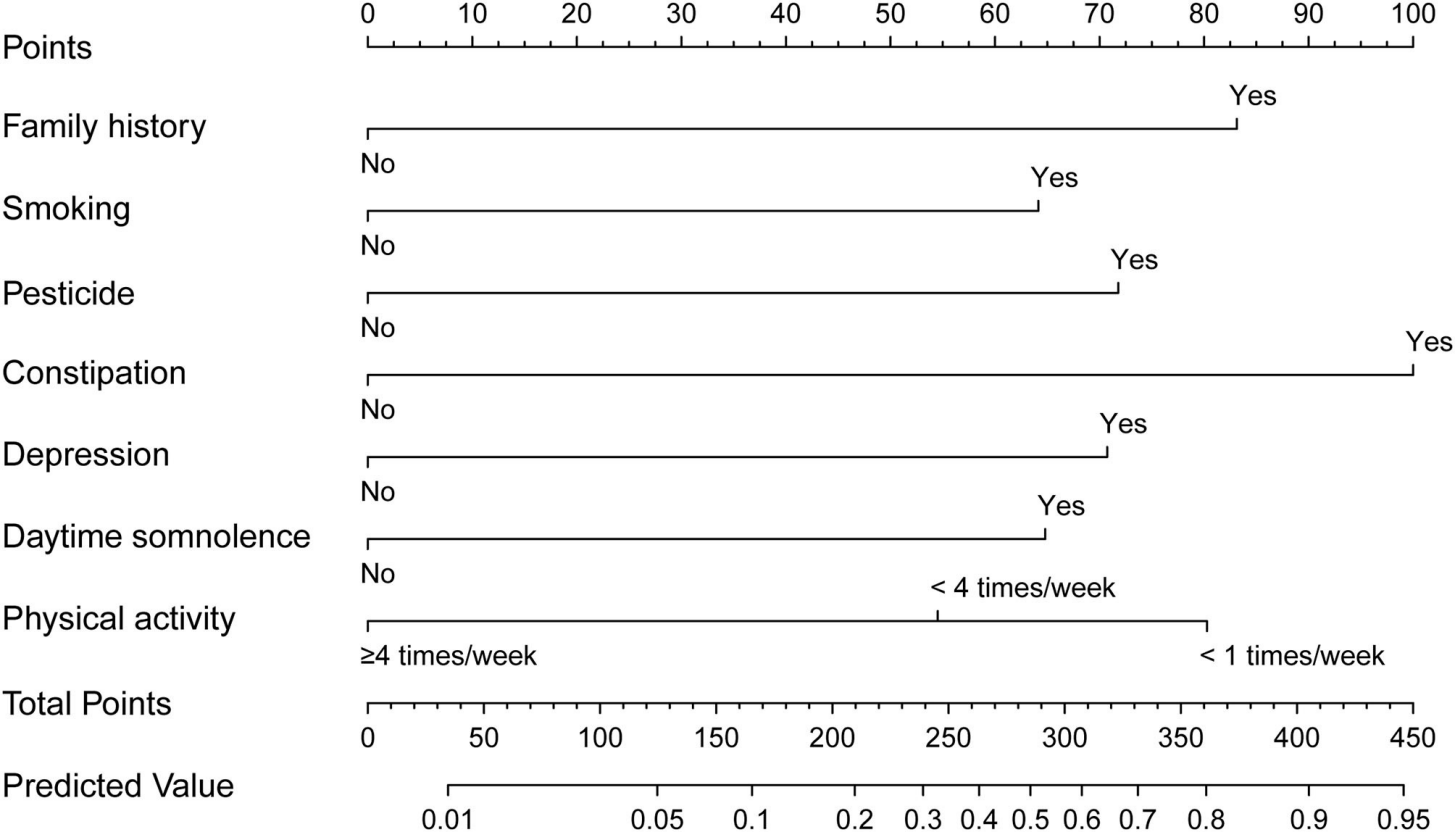
Nomogram to estimate the probability of possible rapid eye movement sleep behavior disorder (pRBD). Find the predictor points on the uppermost point scale that correspond to each subject variable and add them up. The total points projected to the bottom scale indicate the probability of pRBD.

**Figure 3:**
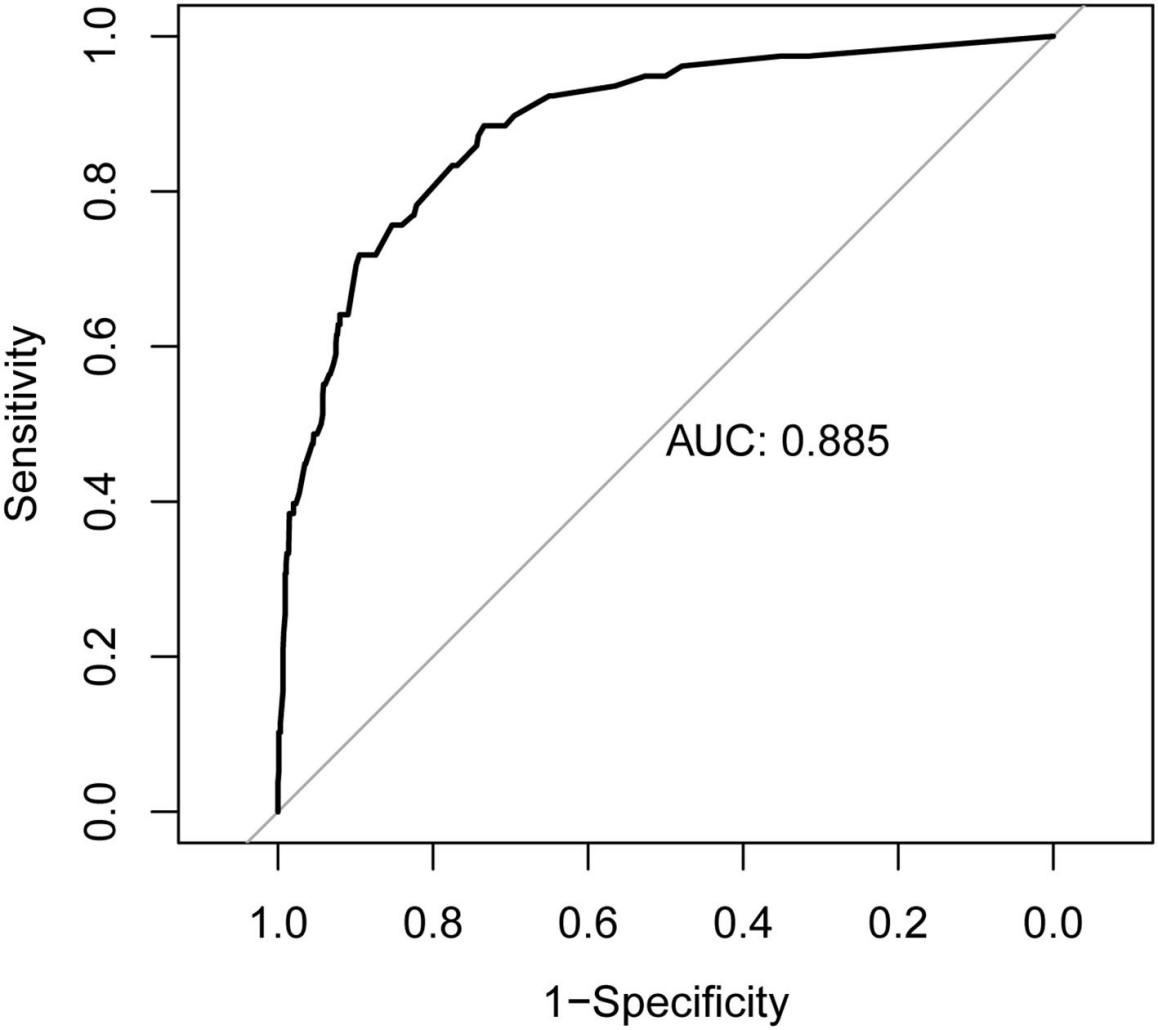
Receiver operating characteristic (ROC) curve of the nomogram. The nomogram had good discriminative power with area under ROC curve of 0.885 (95% CI, 0.845–0.925).

**Figure 4:**
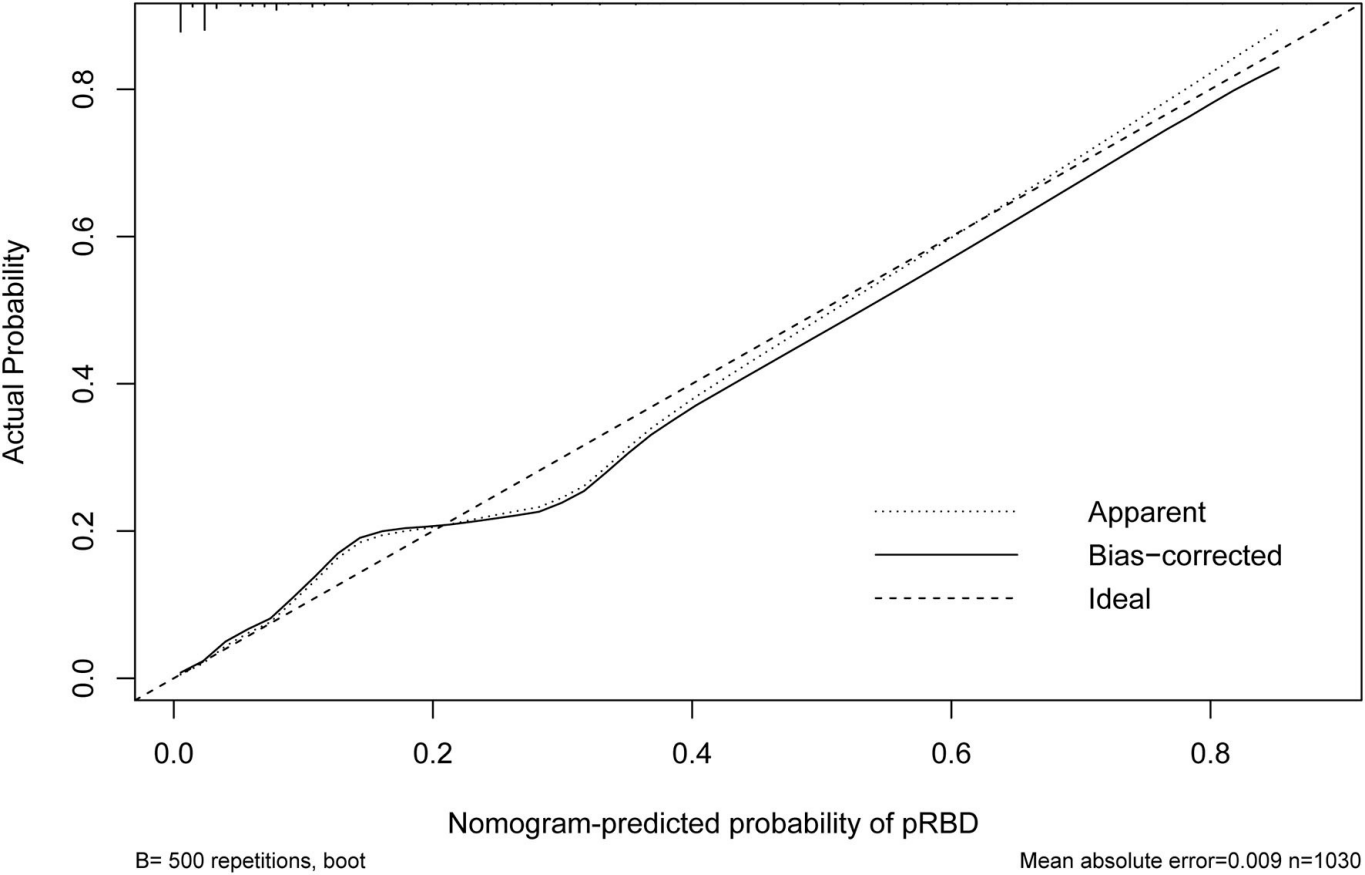
Calibration curve of the nomogram. The X-axis represented the predicted possible rapid eye movement sleep behavior disorder (pRBD) risk. The Y-axis represented the actual diagnosed pRBD. The diagonal dotted line meant a perfect prediction by an ideal model. The short-dashed line represented the apparent prediction of nomogram, and the solid line was bias-corrected by bootstrapping (B = 500 repetitions), indicating observed nomogram performance.

The nomogram is generated and shown in Figure 2. In the nomogram, each value of a variable corresponds to a score, and the corresponding scores for the 7 variables included in the model were summed to achieve a total score for an individual. The total score of an individual was then projected onto a total point scale to obtain the probability of risk of pRBD. The nomogram had high discrimination, with an AUC of 0.885 (95% CI, 0.845–0.925; Figure 3). The optimism-corrected C-index obtained from bootstrap resampling with 1,000 iterations was 0.876, suggesting good internal validation. In addition, the Hosmer-Lemeshow test indicated that the model calibrated well (*p* = 0.980). A calibration curve with 500 bootstrap resamples is presented in Figure 4, which showed that the pRBD probabilities predicted by the nomogram agreed well with the observed probabilities (*p* = 0.956).

### Clinical Utility of Nomogram

**Figure 5:**
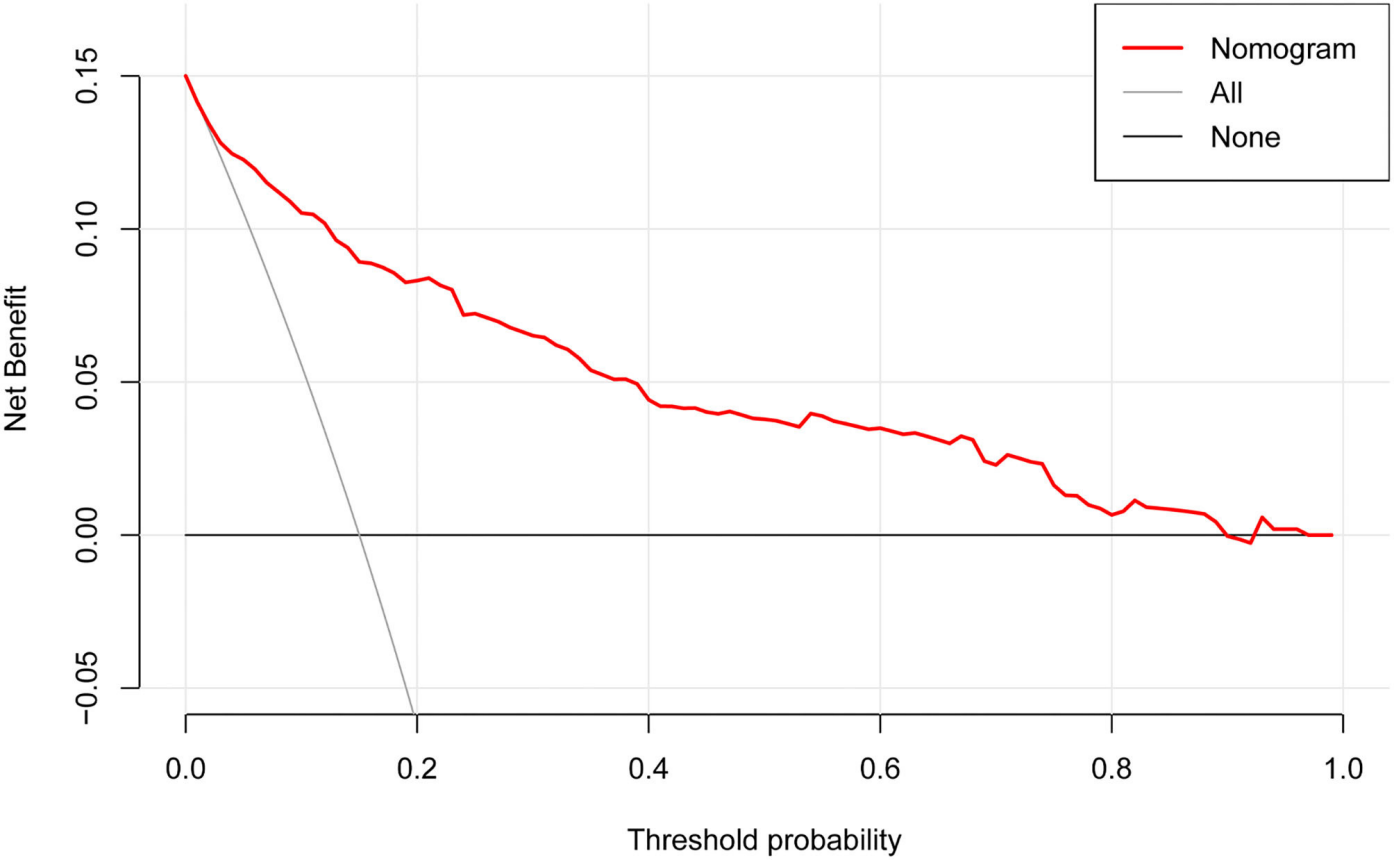
Decision curve analysis for the nomogram. The X-axis showed the threshold probability. The Y-axis measured the net benefit. The red solid line represented the nomogram. The gray solid line represented the assumption that all subjects were possible rapid eye movement sleep behavior disorder (pRBD). The black solid line represents the assumption that no subjects were pRBD.

The DCA for the nomogram is presented in Figure 5. DCA illustrated that the nomogram model has an obvious net benefit for most of the probabilities, especially in threshold probabilities of 10–85%.

## Discussion

The current study has developed a nomogram for predicting pRBD in the community population. It is well discriminated and calibrated for the personal prediction and facilitates individualized treatment. This will improve clinical decisions for clinicians and help patients to get more net benefits. To our knowledge, this study is the first to develop a nomogram for predicting risk for individuals with pRBD. The nomogram visualized the predictive model and made clinical use convenient.

Different from the traditional multivariate logistic regression analysis method, this study adopted the LASSO regression analysis, which performed well in reducing the data dimensionality and decreasing multicollinearity between variables, and it has emerged as a powerful tool under the theory of bias-variance tradeoff in the variable selection (20, 21). Compared with a single predictive factor, which has limited predictive value and is easily affected by multiple factors, the nomogram (a combination of predictive factors) has a tendency for the prediction of higher sensitivity and specificity.

In this study, we found that genetic factors, lifestyles, and environmental exposures are strong predictors of pRBD, which are mostly consistent with previous studies but may differ from those previously defined for neurodegenerative synucleinopathies (11–15, 26). However, the mechanisms underlying these factors in RBD remain largely unclear. We found that a family history of PD or dementia was associated with RBD but this has not been widely reported. Studies have suggested the clustering of the RBD features and increased PD risk and dementia in the iRBD family members (27, 28) and a large population-based study found participants with pRBD were more likely to report a family history of parkinsonism/dementia than controls (11). Genetic studies established that the glucocerebrosidase (GBA) mutations (29) and TMEM175 variant (30) have been associated with both PD and RBD. However, the PD-associated leucine-rich repeat kinase 2 (LRRK2) mutations (31) and microtubule-associated protein tau (MAPT) H2/H1 haplotypes (32) were not associated with RBD, and neither was the AD-related Apolipoprotein E (APOE) ε4 allele (33), suggesting that the genetics of RBD only partially overlaps with PD and DLB. In lifestyles, unlike PD, RBD risk was positively associated with smoking but not with coffee intake, as confirmed in several large epidemiologic studies (14, 15, 26, 34). The explanations for such paradox epidemiological correlations were unclear, but smoking may selectively protect substantia nigra but not other structures involved in synucleinopathies (35), while coffee was infrequently used in the Chinese population. Moreover, a potential protective role for physical activity (≥4 times/week) has been suggested by our study. A recent study suggested that physical inactivity serves as an early and robust prodromal marker of the conversion of iRBD into clinically diagnosed synucleinopathies (36). Further, pesticide exposure is a well-known risk factor for PD and has also been associated with a higher likelihood of RBD in the current and previous studies (26).

We also identified a strong relationship between pRBD and several other non-motor symptoms of PD. There was a positive association between RBD and comorbid depression (37, 38). However, there has been controversy on the role of antidepressants and RBD features, since antidepressants may both treat prodromal depression and trigger early RBD by increasing REM muscle tone and dreaming activity (39). A previous study used the hyperechoic examination of the raphe of the brain stem and substantia nigra to predict the depression in iRBD with a sensitivity of 23.1% and a specificity of 97.1% (40). This finding indicated that serotonergic dorsal raphe dysfunction may be involved in the pathophysiological process of depression in iRBD (40). Our finding that constipation had the strongest relevance with pRBD is consistent with the biopsy studies showing α-synuclein immunostaining in colons of patients with iRBD (41) and with the Braak's hypothesis that α-synuclein pathology may begin with the enteric nervous system (42). Moreover, there has been controversy in the association between RBD and daytime somnolence (36, 43–46), possibly due to the relatively short time period for following up the process of neurodegeneration. In this regard, further prospective studies with a larger sample size, longer follow-up period, and periodic assessment are warranted to determine the association of daytime somnolence with RBD.

Idiopathic RBD, which represents a premonitory symptom of impending neurodegeneration, offers a precious time window, so early identification of pRBD is important to allow neuroprotective and early management strategies to be administered prior to the neurodegenerative diseases. In this study, we used the screened predictors to construct and validate the nomogram model. We found and validated that a combination of family history of PD or dementia, smoking, pesticide exposure, and several non-motor symptoms are useful for predicting pRBD. Since it combines simple and easy-to-collect variables that can easily be gathered in the clinic and communities, the model provides an accurate, non-invasive, low-cost, and rapid method for screening high-risk individuals for pRBD and neurodegenerative disorders. In addition, due to the strength and utility of this nomogram and the easy way to implement, the nomogram is likely to be useful in large population-based primary screening studies.

There are several limitations of this study. First, a major limitation is that the diagnosis of pRBD was completely based on a self-report questionnaire but not the formal PSG confirmation (11–13, 47, 48). As noted in all epidemiological studies, there was a high prevalence of false-positive screen cases as judged by the discrepancy between pRBD and iRBD prevalence. In fact, the prevalence of pRBD (i.e., 7.57%) in our study was similar to those of other population-based studies (i.e., 3.48–7.70%) (49–52) but was higher than those in studies with PSG confirmation (i.e., 0.68–1.15%) (15, 51, 53). Thus, the association of the risk factors in pRBD could only be an approximate correlation and preliminary work for vPSG confirmed iRBD. Considering time and resource consumption, population-based studies on iRBD using PSG are scarce. Therefore, a two-phase screening method that includes an initial questionnaire-based screening for a large population and a PSG-based confirmation for screened positive individuals has been suggested to be an ideal approach (38). Second, the construction of the nomogram based on a cross-sectional study and a relatively small sample size, thus further larger scale studies are needed for validation. We are referring to further video-polysomnographic confirmation and will continue to build the cohort with an expanded sample size. Third, only internal validation was performed. The data for nomogram development and validation are entirely from the community population in Beijing, which could potentially limit the generalizability of the nomogram in other Chinese areas. Last, the success of the model will also rely on the comprehensiveness of the inclusion of the potential risk factors. Therefore, more factors need to be included in future analysis.

In conclusion, the nomogram we developed can successfully predict pRBD risk for elderly subjects. Future studies with larger numbers of participants will be useful for updating and validating this nomogram to improve predictions of pRBD risk among the community population.

## Data Availability Statement

The raw data supporting the conclusions of this article will be made available by the authors, without undue reservation.

## Ethics Statement

The studies involving human participants were reviewed and approved by the Medical Ethics Committee of Xuanwu Hospital of Capital Medical University ([2020]060). The patients/participants provided their written informed consent to participate in this study.

## Author Contributions

HL designed the study, analyzed the data, interpreted the results, and wrote the manuscript. X-YL, JH, WL (4th author), FX, JZ, RH, HW, LC, JY, XL, YS, and WL (15th author) collected the data. XW, ZW, RK, YL, and JX supervised the study. YD and QY designed the study and supervised the study. CW designed the study, supervised the study, and revised the manuscript. All authors contributed to the article and approved the submitted version.

## Funding

This study was supported by grants from the National Natural Science Foundation (81771212 and 82171412), the Ministry of Science and Technology (2016YFC1306000), and the Special Fund from Key Laboratory of Neurodegenerative Diseases, Ministry of Education of China (PXM2019_026283_ 000002).

## Conflict of Interest

The authors declare that the research was conducted in the absence of any commercial or financial relationships that could be construed as a potential conflict of interest.

## Publisher's Note

All claims expressed in this article are solely those of the authors and do not necessarily represent those of their affiliated organizations, or those of the publisher, the editors and the reviewers. Any product that may be evaluated in this article, or claim that may be made by its manufacturer, is not guaranteed or endorsed by the publisher.

## Abbreviations

REM: rapid eye movement
RBD: REM sleep behavior disorder
iRBD: idiopathic RBD
pRBD: possible REM sleep behavior disorder
PSG: polysomnography
vPSG: video-polysomnography
RBDQ-HK: RBD Questionnaire–Hong Kong
PD: Parkinson's disease
DLB: dementia with Lewy bodies
MSA: multiple system atrophy
BMI: body mass index
CI: confidence interval
CO: carbon monoxide
OR: odds ratio
IQRs: interquartile ranges
RSS: residual sum of squares
LASSO: least absolute shrinkage and selection operator
ROC: receiver operating characteristic
AUC: the area under ROC curve
C-index: Concordance index
DCA: decision curve analysis
SCOPA-AUT: scale for outcomes in PD-autonomic.
