# Supplementary material for: Development and Validation of a Predictive Nomogram for Possible REM Sleep Behavior Disorders
Source: Front Neurol. 2022 Jun 29;13:903721. doi: 10.3389/fneur.2022.903721 (PMC9277017; doi:10.3389/fneur.2022.903721)
Supplement: Supplementary file 1 [file Data_Sheet_1.pdf]

Supplementary Material

Assessment of potential risk factors

We classified subjects into three age groups: $<$ 65 years, 65 to 75 years, and $>$75 years (1). Levels of education were categorized as illiteracy, primary school, middle school, and high school and above. Weight and height were measured by investigators. Body mass index (BMI) was calculated as weight (kg)/height (m^2). Subjects with BMI$\geqslant$30 kg/m^2 were considered as obesity, according to WHO-defined international BMI cut-off value (2).

Family History of PD or dementia was assessed by a yes/no question.

Medical histories of hypertension, diabetes, coronary heart disease hyperlipidemia, stroke, general anesthesia were obtained through self-report. For subjects having the above-mentioned diseases, the use of medications (e.g. calcium channel blockers, antidiabetics, aspirin, and statins) was also recorded. For anesthesia, subjects were asked to recall whether they had any general anesthesia before (yes/no).

Smoking: Defined as continuous or cumulative smoking for 6 months or more. Alcohol drinking: Defined as drinking at least once a month. Coffee and tea use: defined as drinking one cup or more per week. The times spent doing exercise were assessed through questionnaire, including (< 1 times, < 4 times and $\geqslant$4 times/week).

History of exposure to pesticides (insecticides and herbicides) was assessed by a yes/no question. Occupational exposure to toxicants: Defined as work exposure to the materials for more than 6 months, including organic solvent, heavy metals, were assessed by a yes/no question. Histories of head injury and CO poisoning: Defined as head injury and CO poisoning with loss of consciousness.

Scales for assessing the motor and non-motor symptoms: Standardized motor examination: Assessed with the Movement Disorders Society Unified Parkinson Disease Rating Scale part III (MDS-UPDRS- III) (3). Olfactory dysfunction: Using Hyposmia rating scale (HRS), and a cut-off value of 23 on the HRS score (4). Constipation: Using Rome III criteria, and in accordance with 2 or more items diagnosed as functional constipation (5). Cognitive impairment: Using the Mini-Mental State Examination (MMSE). MMSE score cut-off value of 17 (illiteracy), 20 (primary school education), 23 (secondary school education) of 30 points (6). Depression: Using the 17-item Hamilton Depression Scale (HAMD), and a cut-off value of 8 on the HAMD score (7). Daytime somnolence: Using Epworth Sleepiness scale (ESS), and a cut-off value of 10 on the ESS score (8). Autonomic symptoms, assessed by the scale for outcomes in PD-autonomic (SCOPA-AUT) (9).

REFERENCES

1. Zhang H, Gu Z, Yao C, Cai Y, Li Y, Mao W, et al. Risk factors for possible REM sleep behavior disorders: A community-based study in Beijing. Neurology. (2020) 95:e2214-e24. doi: 10.1212 /WNL.0000000000010610
2. World Health Organization. Obesity: Preventing and Managing the Global Epidemic. *Geneva: World Health Organization*. (2000)
3. Goetz CG, Tilley BC, Shaftman SR, Stebbins GT, Fahn S, Martinez-Martin P, et al. Movement Disorder Society-sponsored revision of the Unified Parkinson's Disease Rating Scale (MDS-UPDRS): scale presentation and clinimetric testing results. *Mov Disord*. (2008) 23:2129–70. doi: 10.1002/mds.22340
4. Millar Vernetti P, Perez Lloret S, Rossi M, Cerquetti D, Merello M. Validation of a new scale to assess olfactory dysfunction in patients with Parkinson's disease. *Parkinsonism Relat Disord*. (2012) 18:358–61. doi: 10.1016/j.parkreldis.2011.12.001
5. Drossman DA, Corazziari E, Delvaux M. Rome III and the Functional Gastrointestinal Disorders. *3 rd edition*. (2006)
6. Folstein MF, Folstein SE, McHugh PR. "Mini-mental state". A practical method for grading the cognitive state of patients for the clinician. *J Psychiatr Res*. (1975) 12:189–98. doi: 10.1016/0022-3956(75)90026-6
7. Zimmerman M, Martinez JH, Young D, Chelminski I, Dalrymple K. Severity classification on the Hamilton depression rating scale. *J Affect Disord*. (2013) 150:384-8. doi: 10.1016/j.jad.2013.04.028
8. Johns MW. A new method for measuring daytime sleepiness: the Epworth sleepiness scale. *Sleep*. (1991) 14:540–5. doi: 10.1093/sleep/14.6.540
9. Visser M, Marinus J, Stiggelbout AM, Van Hilten JJ. Assessment of autonomic dysfunction in Parkinson's disease: the SCOPA-AUT. *Mov Disord.* (2004) 19:1306-12. doi:10.1002/mds.20153

TABLE S1 | The English version of RBDQ-HK

RBD Questionnaire–Hong Kong (RBDQ-HK)								
Symptoms	Lifetime occurrence			Recent 1-year frequency				
	I don't remember/ I don't know	No	Yes	Did it happen in the recent 1-year?	Once or few times per year	Once or few times per month	1–2 times per week	3 times or above per weeks
1. Did you often have dreams?				☐Yes ☐ No (Please go to the next question)				
2. Did you often have nightmares?				☐Yes ☐ No (Please go to the next question)				
3. Did you have dreams with an emotional and sorrowful content?				☐Yes ☐ No (Please go to the next question)				
4. Did you have dreams with a violent or aggressive content (e.g.,fighting with someone)?				☐Yes ☐ No (Please go to the next question)				
5. Did you have dreams with a frightening and horrifying content (e.g., being chased by ghost)?				☐Yes ☐ No (Please go to the next question)				
6. Did you have sleep talking?				☐Yes ☐ No (Please go to the next question)				
7. Did you shout, yell or swear during your sleep?				☐Yes ☐ No (Please go to the next question)				
8. Did you move your arms or legs in response to your dream contents during sleep?				☐Yes ☐ No (Please go to the next question)				
9. Have you ever fallen from your bed?				☐Yes ☐ No (Please go to the next question)				
10. Have you ever hurt yourself or your bed-partner while you were sleeping?				☐Yes ☐ No (Please go to the next question)				
11. Have you ever attempted to assault your bed-partner or almost hurt yourself while you were sleeping?				☐Yes ☐ No (Please go to the next question)				
12. Did the scenario				☐Yes				

described in 10 or 11 relate to your dream contents?				☐ No (Please go to the next question)				
13. Did the situations described above disturb your sleep?				☐Yes<br>☐ No				

Note. For each lifetime item, Q1–Q5 and Q13 were scored as follows: ‘‘don’t know” = 0, ‘‘no” = 0, ‘‘yes” = 1; Q6–Q12 were additionally weighted: ‘‘don’t know” = 0, ‘‘no” = 0, ‘‘yes” = 2. For each recent 1-year frequency item with a five-point scale, Q1–Q5 and Q13 were scored as follows: ‘‘no” = 0,‘‘yes/once or few times per year” = 1,‘‘once or few times per month” = 2,‘‘1–2 times per week” = 3,‘‘3 times or above per week” = 4; Q6–Q12 were additionally weighted: ‘‘no” = 0,‘‘yes/once or few times per year” = 2,‘‘once or few times per month” = 4,‘‘1–2 times per week” = 6,‘‘3 times or above per week” = 8. The total RBDQ-HK score was calculated by the sum of the scores of all lifetime items and recent 1-year frequency items. The total RBDQ-HK score can be ranged from 0 to 100.

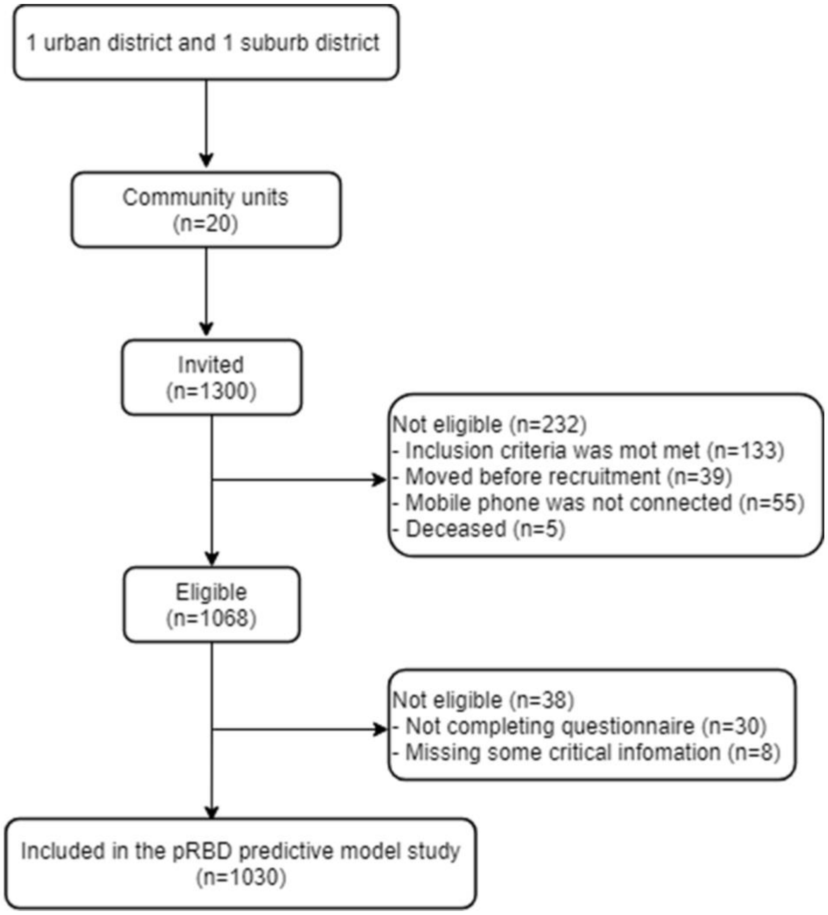

FIGURE S1 | Flow chart of epidemiological design
